# Open-source versus proprietary large language models for academic writing in medicine: A controlled evaluation of DeepSeek-R1 and ChatGPT-o1 in neuroradiology

**DOI:** 10.1177/20552076261464745

**Published:** 2026-09-02

**Authors:** Semil Eminovic, Bogdan Levita, Robin Schmidt, Maximilian Lindholz, Dmitriy Desser, Tobias Penzkofer, Mike P. Wattjes, Jawed Nawabi

**Affiliations:** 1 Department of Radiology, Berlin Institute of Health, Charité – Universitätsmedizin Berlin, Humboldt-Universität zu Berlin, Freie Universität Berlin, Berlin, Germany; 2 Department of Neuroradiology, Berlin Institute of Health, Charité – Universitätsmedizin Berlin, Humboldt-Universität zu Berlin, Freie Universität Berlin, Berlin, Germany

**Keywords:** natural language processing, artificial intelligence, publishing/standards, reproducibility of results, neuroradiology

## Abstract

**Objectives:**

Large language models (LLMs) are being used to facilitate academic writing. We aimed (1) to assess the quality, integrity, and factual reliability of LLM-assisted academic writing in medicine, and (2) to compare the performance of open-source versus proprietary LLMs, reflecting differences in model transparency.

**Methods:**

In this prospective, controlled evaluation study, an open-source model (DeepSeek-R1) and proprietary model (ChatGPT-o1) completed ten academic tasks: generating five scientific essays and five evidence-based question-answers covering clinical topics in neuroradiology. Two radiologists rated outputs using 14 Likert and 7 binary criteria across the domains of academic quality, linguistic expression, factual reliability. Paired Wilcoxon/McNemar (Holm) assessed differences; ICC/Cohen’s κ assessed reliability.

**Results:**

DeepSeek-R1 achieved higher overall mean Likert scores than ChatGPT-o1 (mean ratings ± standard deviation: 3.23 ± 0.44 vs 3.02 ± 0.30, p = 0.021). It significantly outperformed in reasoning depth (p = 0.015), contextual coherence (p = 0.043), subtlety (p = 0.037), and evidence integration (p = 0.027). Although both models cited sources in every output, citation reliability was poor. DeepSeek-R1 cited more real publications (55.1% vs. 33.3%, p=0.053) but showed a higher confirmed fabrication rate (36.7% vs. 2.6%, p<0.001). DeepSeek-R1 respected word limits in 90%, ChatGPT-o1 only in 50%. Neither reviewer reliably noticed AI‐generated text features. Inter-rater reliability was poor for Likert criteria (ICC = 0.466) and substantial for binary items (κ = 0.746).

**Conclusion:**

DeepSeek-R1 outperformed ChatGPT-o1 in generating academic content for neuroradiology, highlighting the potential of transparent, locally deployable models. However, moderate quality, citation errors and hallucinations indicate that LLMs are not yet sufficient for unsupervised academic writing and demand rigorous human review.

## Relevance statement

Large language models (LLMs) are increasingly used to support academic writing in medicine, but their outputs raise concerns about quality, reliability, and adherence to good scientific practice. In a controlled evaluation of ten academic tasks in neuroradiology, we compared an open-source LLM (DeepSeek-R1) with a proprietary model (ChatGPT-o1). Our findings demonstrate consistent but bounded performance advantages for the open-source model, persistent and severe citation unreliability across both systems, and inconsistent detectability of AI-generated content. These findings may inform practices of journal editors (citation verification of AI-assisted submissions), academic institutions (governance frameworks and AI literacy curricula), and researchers (the role of open-source, locally deployable models for sensitive medical content) navigating the responsible integration of LLMs into scientific workflows.

## Key points

• **Key Point 1** DeepSeek-R1 outperformed ChatGPT-o1 in overall quality (mean Likert score 3.23 ± 0.44 vs. 3.02 ± 0.30, p = 0.021). It significantly outperformed in key domains: reasoning depth (p = 0.015), contextual coherence (p = 0.043), subtlety (p = 0.037), and evidence integration (p = 0.027).
• **Key Point 2** Citation reliability was poor in both models. Most references were inaccurate or hallucinated despite appearing in all outputs.
• **Key Point 3** AI-generated features went unnoticed: reviewers could not reliably distinguish human-like text from model outputs.
• **Key Point 4** Practical considerations arising from these findings include citation-level verification of AI-assisted submissions, institutional evaluation of open-source LLMs for sensitive medical contexts, and human oversight prior to publication of AI-assisted content.

## Introduction

Large language models (LLMs) have achieved a level of fluency and coherence that makes their outputs increasingly indistinguishable from those by humans.^1,2^ This development has raised critical concerns in academic settings, particularly related to authorship, originality, and integrity. In medicine and other high-stake disciplines, these concerns are amplified, as accuracy, authenticity, and trust in written content are essential for both education and clinical decision-making. A recent nationwide survey in Germany revealed that nearly two-thirds of university students already use Artificial Intelligence (AI)-based tools in their studies, with ChatGPT being most commonly used.^3^ While these tools promise convenience and efficiency, they also introduce new risks: ChatGPT can produce inaccurate or incomplete information, potentially leading to misunderstandings of complex scientific concepts.^4^ Furthermore, the indistinguishability of AI-generated writing has given rise to ethical challenges, including academic dishonesty and plagiarism.^5,6^ At the same time, academic and clinical institutions are facing a second, structural dilemma: whether to depend on closed-source, proprietary models or to invest in the development and evaluation of open-source alternatives. While proprietary models demonstrate strong general performance, their use is hindered by limitations in transparency, customizability, data privacy, and reproducibility.^7,8^ These constraints are particularly problematic in high-stakes domains like medicine, where clinicians and educators must be able to audit model behavior, adapt systems to domain-specific tasks, and maintain compliance with regulatory standards. In contrast, open-source LLMs are emerging as a promising alternative. These models offer full access to weights and architecture, enabling local deployment, fine-tuning on institutional data, and alignment with privacy and governance requirements. Moreover, open-source development fosters reproducibility, community-driven improvements, and equitable access.^7,8^ However, open-source LLMs remain insufficiently validated in academic medicine, especially in specialized fields such as neuroradiology, where both technical precision and clinical nuance are required.

This study aims to assess both the practical performance and the implications for good scientific practice of LLM-generated content in academic medicine, as well as the role that open-source solutions may play in promoting responsible use. To address this, we examined two converging challenges. First, we evaluate the performance and perceptual limitations of LLM-generated academic content in a specialized medical domain. Specifically, we assessed whether medical experts can identify AI-generated text in neuroradiology and rate its academic quality, linguistic expression, and content integrity. Second, we investigated how an open-source model (DeepSeek-R1) performs compared to a proprietary model (ChatGPT-o1) in generating domain-specific academic content within neuroradiology.

## Methods

### General study design

Due to the human-generated sample dataset, it was not mandatory to obtain a positive ethics vote from Charité’s Ethics Committee. In this prospective, controlled evaluation study conducted between February and March 2025 (all prompting was conducted on February 18, 2025), two state-of-the-art large language models, DeepSeek-R1 (*DeepSeek*) and ChatGPT-o1 (*OpenAI*), were each assigned ten identical question-based tasks designed to generate domain-specific academic content in neuroradiology. DeepSeek-R1 and ChatGPT-o1 were selected based on their state-of-the-art performance in both general-purpose reasoning tasks and domain-specific applications.^9^ Both LLMs employ “chain-of-thought”-reasoning, that guides to models to follow and “deep think” a reasoning process when dealing with complex problems.^8,10^ LLM-generated responses by both models were independently evaluated by two radiologists (J.N.: board certified diagnostic and interventional neuroradiologist with both eight years of clinical and research experience; S.E.: third-year radiology resident with more than five years research experience). For the purpose of this study, we operationally defined an academic writing task as a domain-specific text generation task that requires (i) structured presentation of medical or scientific content, (ii) integration of external evidence with appropriate citation, (iii) adherence to formal length and stylistic conventions of academic medicine, and (iv) demonstration of analytical reasoning rather than pure factual recall. We deliberately selected two distinct sub-formats to capture different facets of this construct: long-form discursive essays (testing argumentation, synthesis, and stylistic competence) and short-form, evidence-based Q&A items (testing guideline integration, citation reliability, and conciseness).

### Question design and prompting

Recognizing that LLM performance can vary with prompt length, complexity, and task type, we included two medical academic formats: five long-form essay tasks and five short-form, evidence-based question–answer (Q&A) items. Essays were designed to elicit discursive writing on broader scientific or ethical topics in neuroradiology, while Q&A tasks targeted more specific, guideline-oriented clinical questions to assess the integration of structured medical knowledge. To enhance generalizability, a heterogeneous set of topics were curated, covering both clinical and translational aspects of neuroradiology. These ranged from AI-based diagnostics and neuro-oncology to clinical imaging guidelines and ethical considerations, Table 1. ChatGPT was used solely for initial topic pool generation; final topic selection and refinement was performed independently by the authors (J.N. and S.E.) based on clinical and research relevance. All selected topics correspond to well-established neuroradiology subject areas. To ensure consistency across tasks, all prompts were carefully structured with clear instructions, length requirements, and domain-specific expectations. Representative examples are provided below for both essay and Q&A formats.

**Table 1.** Essay and Q&A topics used to evaluate LLM performance in neuroradiology.

Essay Writing	Evidence-based Q&A
AI-supported MRI analysis for detecting neurodegenerative diseases	Current guidelines for imaging in acute stroke diagnosis
Radiomics and machine learning in brain tumor diagnosis	MRI guidelines for diagnosing multiple sclerosis
The role of functional MRI (fMRI) in pre-surgical epilepsy diagnosis	Indications for MR spectroscopy in brain tumor diagnostics
A comparison of CT perfusion imaging versus MRI in ischemic stroke	Diffusion-weighted imaging (DWI) recommendations for stroke diagnosis
Ethical and data-protection challenges of AI in neuroradiology.	PET/MRI recommendations for Alzheimer’s disease diagnosis.

#### Prompt example for essay writing

*“Write a scientific essay (around 1000 words) on the topic: 'AI-Supported MRI Analysis for Detecting Neurodegenerative Diseases'. Structure the essay into an introduction, main body, and conclusion. Explain the role of AI in detecting Alzheimer's, Parkinson's, and Multiple Sclerosis. Analyze recent studies (from 2020 onwards) and discuss benefits and ethical challenges. Use factual, scientific language and cite relevant sources in APA style. Ensure that the text has a logical structure and that the arguments are supported by examples?”*

#### Prompt example for an evidence-based Q&A

“*Answer the following evidence-based question: Question: What are the current guidelines for imaging in acute stroke diagnosis according to the American Heart Association (AHA) and American Stroke Association (ASA) or comparable professional societies?*

#### Answer requirements

*• State the recommended imaging modalities and their purpose (e.g., CT, CTA, CTP, MRI).*
*• Describe the indications for perfusion imaging and large vessel occlusion detection.*
*• Mention the time windows for different imaging methods (e.g., early CT vs. advanced MRI for late presentation).*
*• Refer to the latest guidelines.*
*• Summarize your answer in a maximum of 200 words.*
*• Cite the source (e.g., AHA guidelines, year) in APA style.”*

Both models were accessed via their respective public web interfaces (DeepSeek-R1, version R1 released January 2025, chat.deepseek.com; ChatGPT-o1, version o1 released December 2024, chat.openai.com) on 18 February 2025. Parameters such as temperature, top-p, and system prompts were not user-configurable and remained at providers’ default settings. All prompts were submitted using identical phrasing as standalone queries in fresh chat sessions, with the cache cleared between queries. The complete set of all ten prompts is provided in Supplementary Text 1 (essay prompts) and Supplementary Text 2 (Q&A prompts). All corresponding model responses are documented and available on request.

### Response evaluation

**Figure 1.**
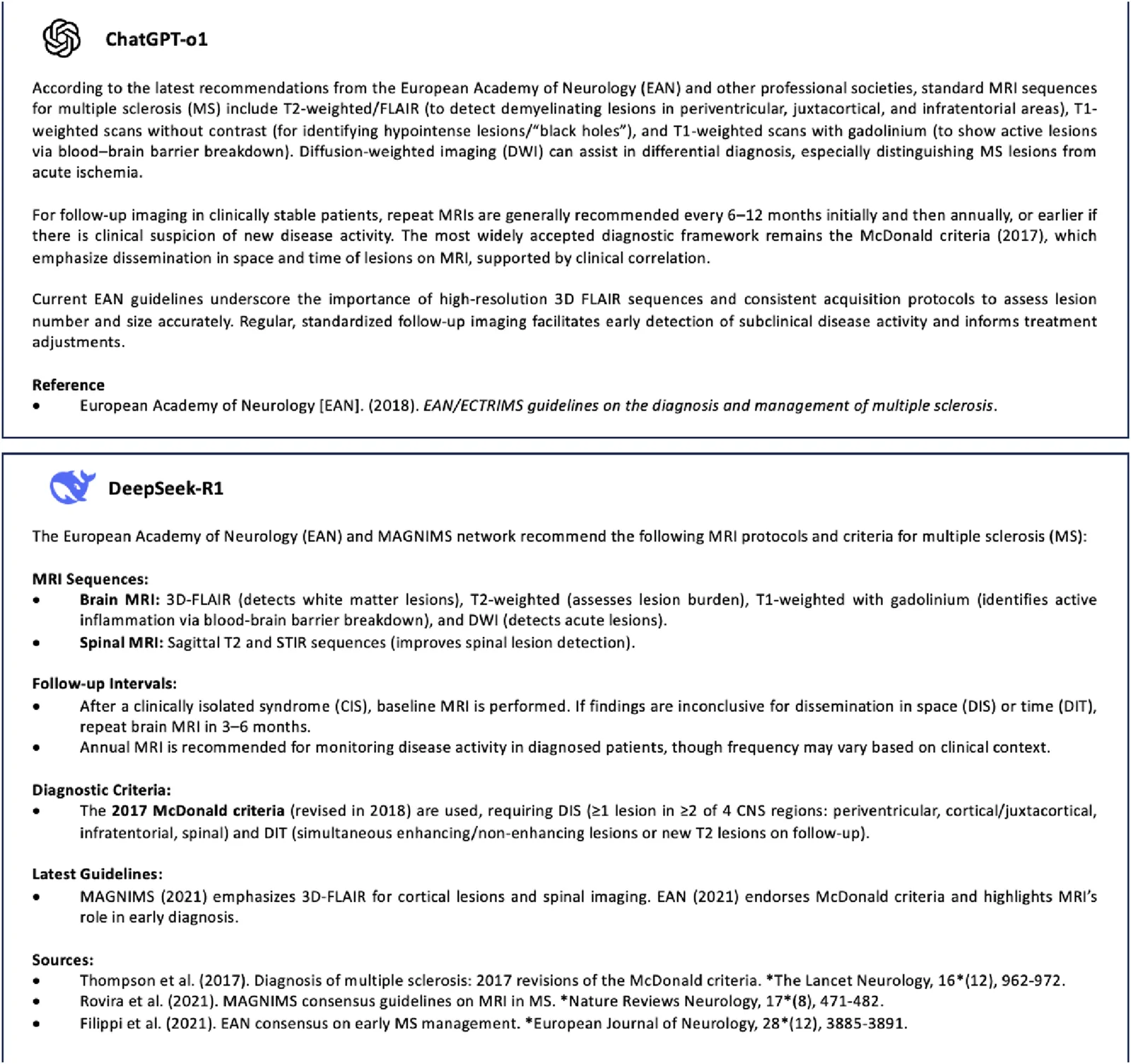
Exemplary display of the Q&A response on ‘MRI Guidelines for Diagnosing Multiple Sclerosis‘ for both ChatGPT-o1 and DeepSeek-R1.

All 20 outputs were de-identified using randomized alphanumeric identifiers prior to rating. Presentation order was randomized per task. The blinding key was held by a non-rating co-author and released only after independent completion of all evaluations. Responses were blind-scored independently by using two complementary rating schemes: a 5-point Likert scale applied to 14 qualitative criteria and a binary (yes/no) scale applied to 7 objective criteria. This evaluation framework was developed a priori by two of the authors (S.E., J.N.) and remained unmodified throughout rating. As no validated evaluation framework for LLM-generated academic medical writing currently exists, the criterion set was constructed to span three conceptually distinct domains (academic and analytical quality, linguistic expression, and factual reliability) selected to reflect core demands of evidence-based scientific writing in medicine. Full definitions of the rating criteria are provided in Supplementary Table S1. The criteria were designed to capture distinct dimensions of text quality and to minimize conceptual overlap. Figure 1 displays exemplary Q&A responses on ‘MRI Guidelines for Diagnosing Multiple Sclerosis‘ for both ChatGPT-o1 and DeepSeek-R1.

Likert‐Scale Criteria (n = 14). These items were rated on a 5-point scale (1 = Poor, 5 = Excellent), capturing key qualitative dimensions of academic, linguistic, stylistic, and content-related qualities.

1. Academic Quality – Structure, clarity, and academic rigor
2. Nuance – Subtle distinctions and contextual depth
3. Reasoning Depth – Logical coherence and evidence support
4. Bias Detection – Neutrality and balance in medical content
5. Readability – Accessibility for the target audience
6. Natural Language Flow – Fluidity and sentence‐structure variety
7. Authenticity of Voice – Presence of a distinct, personal writing style
8. Use of Idiomatic & Context-Specific Language – Effective integration of idioms and domain terms
9. Rhetorical Devices & Emotional Nuance – Use of metaphors, analogies, and subtle affect
10. Variability & Redundancy – Diversity of expression and avoidance of repetition
11. Contextual Adaptation & Coherence – Logical progression and adaptation to the question’s context
12. Subtlety in Argumentation – Sophistication and layering of perspectives
13. Citation Accuracy – Correctness and completeness of reference details
14. Evidence Integration – Thoroughness and fidelity of supporting evidence

Binary Criteria (n = 7): These were scored as Yes (1) or No (0), focusing on objective aspects of academic quality, including factual integrity, formal compliance, and the detectability of AI-generated content.

1. Evidence-based – Claims are supported by real, verifiable references
2. Quality – References originate from high-quality (e.g. peer-reviewed) sources
3. Overall Preference – Rater’s choice between LLM 1 and LLM 2
4. Word Limit Respected – Response adheres to the prescribed length
5. AI Authorship Indicator – Presence of stylistic or mechanical markers of AI
6. Factual Evidence Verification – Every reference is factually correct and verifiable
7. Academic/Clinical Suitability – Appropriate rigor for academic or clinical use

For the criterion ‘Readability’, the target audience was defined a priori as a clinically trained reader with working knowledge of neuroimaging terminology. Each cited reference was additionally classified as (a) fully verifiable, (b) partially correct (real publication with ≥1 incorrect detail), (c) unverifiable (not locatable via PubMed, Google Scholar, or DOI lookup), or (d) fully fabricated (DOI resolving to a different publication), using PubMed and Google Scholar as the gold standard (Supplementary Table S2).

### Statistical analysis

The study employed a repeated-measures (paired) design in which two radiologists (J.N. and S.E.) rated each task on 14 Likert criteria (1–5) and 7 binary criteria (yes = 1/no = 0). For each task and criterion, we averaged the two Likert scores, and for the binary ratings we rounded the mean to the nearest integer (≥0.5 → 1, < 0.5 → 0). Paired Likert means were compared with two-sided Wilcoxon signed-rank tests (14 tests) and paired binary outcomes with McNemar’s tests (7 tests). Effect sizes for paired Wilcoxon signed-rank tests were calculated using the matched-pairs rank-biserial correlation (r_rb), with values of 0.1, 0.3, and 0.5 representing small, medium, and large effects, respectively. Because of the exploratory study design and limited number of paired tasks, unadjusted p-values were used as the primary inferential measure, while Holm correction was additionally explored to assess the robustness of findings against multiple testing. Stratified analyses were additionally performed separately for essay tasks (n = 5 pairs) and Q&A tasks (n = 5 pairs) using the same paired Wilcoxon signed-rank procedure with Holm correction and exact p-values (Supplementary Tables S4, S5). We reported descriptive statistics (mean ± standard deviation) for each criterion across tasks and models. Citation category distributions were compared between models using the chi-squared test, fabrication and verifiability rates were compared using Fisher’s exact test. Inter-rater reliability was assessed by computing ICC(2,1) from a two-way random-effects, absolute-agreement model on the mean Likert scores suiting continuous/ordinal data and by calculating Cohen’s κ^11^ on the binary ratings quantifying agreement for categorical outcomes. Per-task overall mean Likert scores were computed across all 14 criteria to assess whether individual tasks disproportionately influenced overall results (Supplementary Table S8). All analyses were conducted in Python 3.9.13 (Pandas 2.2.2, NumPy 1.23.1, SciPy 1.13.1, statsmodels 0.14.4, Matplotlib 3.9.2, Seaborn 0.13.2, Scikit-learn 1.5.2, OpenPyXL (Excel) 3.1.5).

## Results

**Figure 2.**
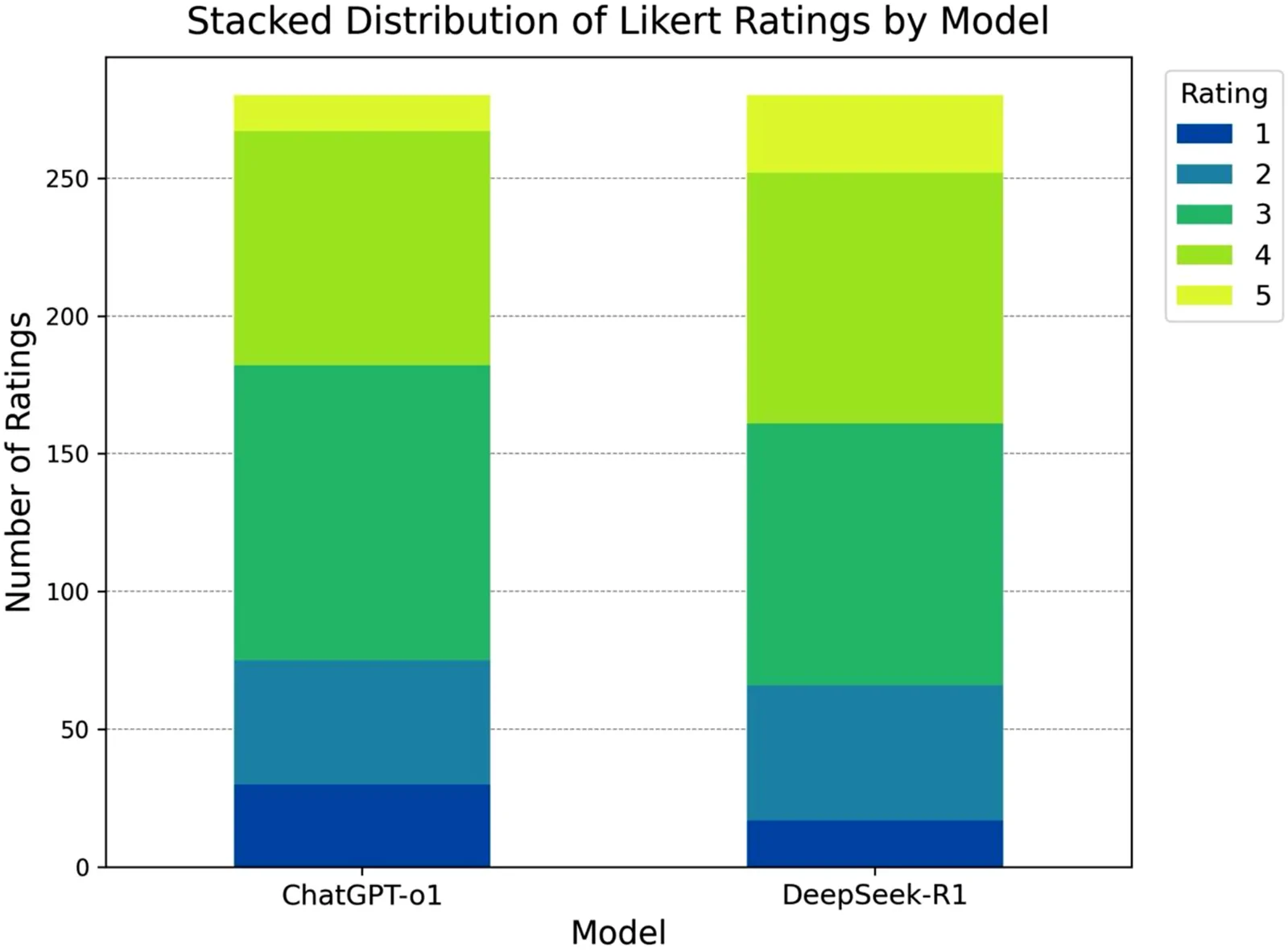
Stacked barplots show the overall distribution of Likert ratings for all Likert-rated criteria DeepSeek-R1 and OpenAI o1 (each model received n = 280 ratings). Compared across all 280 ratings, DeepSeek-R1 received fewer ‘1’ ratings (6.1% vs. 10.7%), slightly more ‘2’ ratings (17.5% vs. 16.1%), fewer ‘3’ ratings (33.9% vs. 38.2%), slightly more ‘4’ ratings (32.5% vs. 30.4%), and notably more ‘5’ ratings (10.0% vs. 4.6%) than ChatGPT-o1.

**Figure 3.**
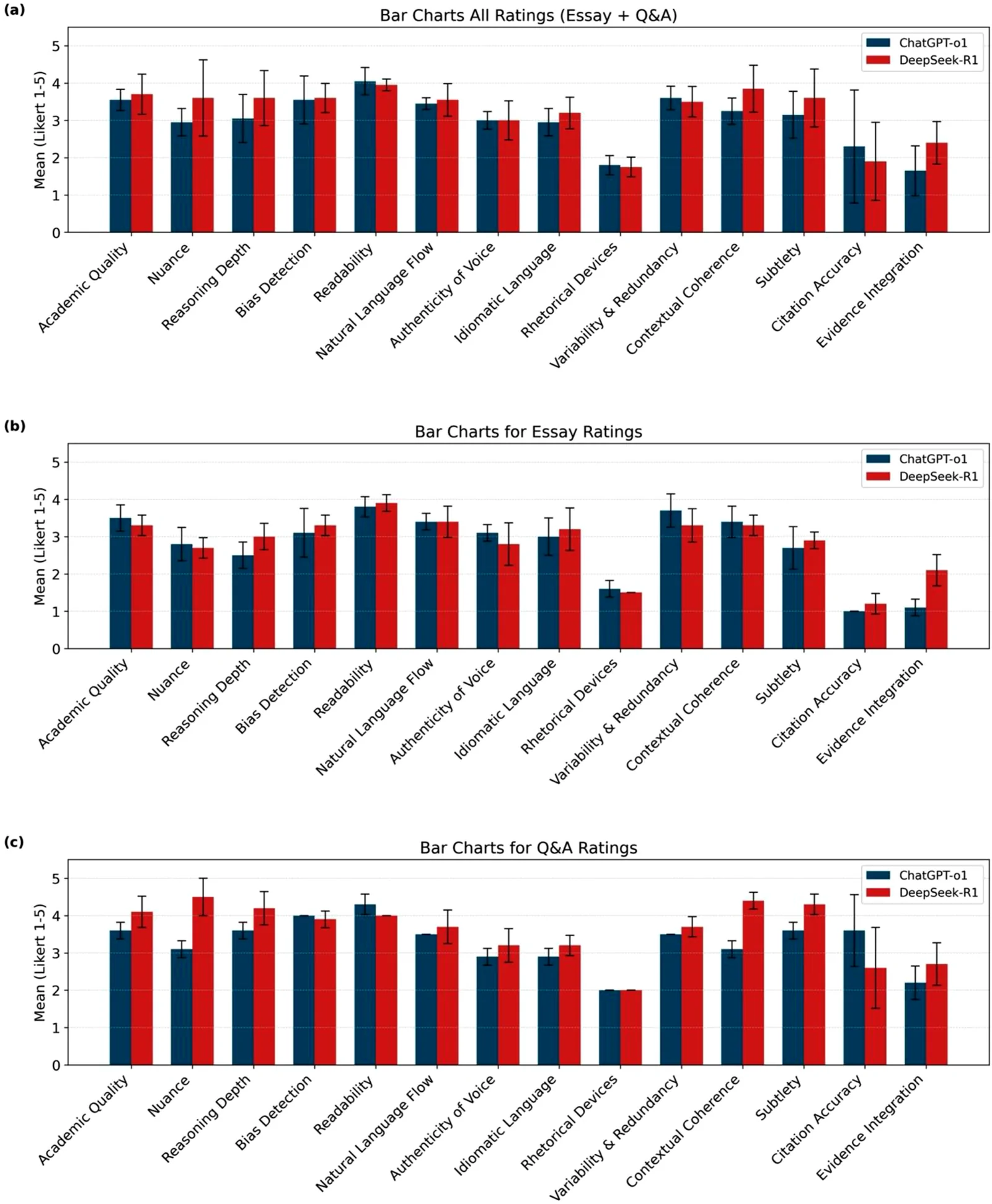
Grouped bar plots comparing mean Likert ratings (±SD) of ChatGPT-o1 and DeepSeek-R1 across all evaluation criteria for Essays and Q&A combined (a), Essays only (b), and Q&A only (c). Error bars represent ± 1 standard deviation.

**Figure 4.**
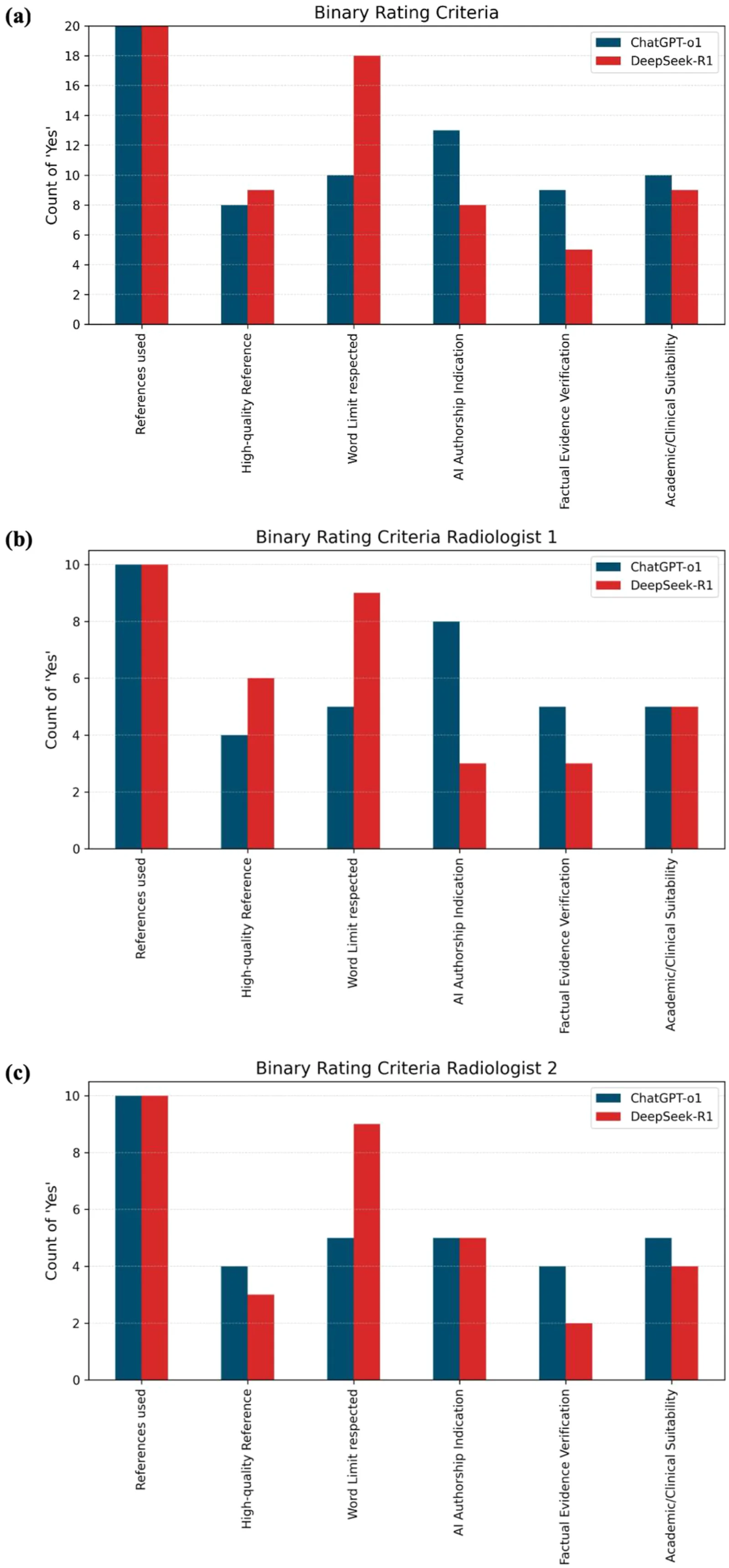
Bar chart comparing the count of positive (“Yes”) ratings for each binary evaluation criterion between ChatGPT-o1 (blue) and DeepSeek-R1 (red) (a) overall for both radiologists, (b) for radiologist 1 and (c) radiologist 2.

Across all 14 Likert‐scale criteria for both essays and Q&A, DeepSeek-R1 achieved consistently higher mean ratings than ChatGPT-o1 (overall mean ratings ± standard deviation (SD): DeepSeek-R1 3.23 ± 0.44 versus ChatGPT-o1 3.02 ± 0.30, p = 0.021*). Figure 2 displays the overall distribution of Likert ratings for both models demonstrating that DeepSeek-R1 received more top ‘5’ ratings (n1 = 28 vs. n2 = 13) and lower number of ‘1’ ratings (n1 = 17 vs. 30). Significant advantages for DeepSeek-R1 were observed in Reasoning Depth (p = 0.015*), Contextual Coherence (p = 0.043*), Subtlety (p = 0.037*) and *Evidence Integration *(p = 0.027*) (Table 2, Figure 3). Several domains additionally demonstrated large effect sizes favoring DeepSeek-R1, including Reasoning Depth (r_rb = 1.00), Contextual Coherence (r_rb = 0.90), Subtlety (r_rb = 0.86), and Evidence Integration (r_rb = 0.78). After Holm correction for 14 simultaneous comparisons, no individual criterion retained statistical significance (all p_Holm > 0.05; Supplementary Table S3), consistent with the study’s limited power to detect effects below the large-effect threshold under conservative multiplicity adjustment. The remaining criteria trended in DeepSeek-R1’s favor but did not reach statistical significance. Both DeepSeek-R1 and ChatGPT-o1 scored high on Readability (3.95 ± 0.16 vs. 4.05 ± 0.37) yet performed poorly in deploying Rhetorical Devices (1.75 ± 0.26 vs. 1.80 ± 0.26) and in *Citation Accuracy* (1.90 ± 1.05 vs. 2.30 ± 1.51). A granular per-reference citation analysis is provided in Supplementary Table S2. ChatGPT-o1 (n=39) predominantly produced unverifiable references (64.1%), whereas DeepSeek-R1 (n=49) showed a substantially higher confirmed fabrication rate (36.7% vs. 2.6%, Fisher’s exact p<0.001). Overall category distributions differed significantly (χ^2^=35.98, df=3, p<0.001), fully verifiable rates were similar (20.5% vs. 22.4%, p=1.00). In binary assessments, DeepSeek-R1 respected the word limit 90% of the time versus 50% for ChatGPT-o1 (Figure 4). In 50% of cases, reviewers identified typical AI stylistic markers in ChatGPT-o1 outputs (e.g. parenthetical clauses are inserted quite frequently), compared to only 10% for DeepSeek-R1.

**Table 2.** Mean ratings and statistical comparison between DeepSeek-R1 and ChatGPT-o1 across all evaluation criteria for Likert-based evaluations for both Essays and Q&A using Wilcoxon Paired Test (unadjusted p-values; Holm-corrected p-values in Supplementary Table S3; n = 14 rating criteria, n = 5 Essays, n = 5 Q&As, n = 2 rater).

Rating criteria	DeepSeek-R1	ChatGPT-o1	Wilcoxon paired test
	Mean ± SD	Mean ± SD	P-Value
Overall (all criteria)	3.23 ± 0.44	3.02 ± 0.30	0.021*
Academic Quality	3.70 ± 0.54	3.55 ± 0.28	0.380 (n.s)
Nuance	3.60 ± 1.02	2.95 ± 0.37	0.065 (n.s)
Reasoning Depth	3.60 ± 0.74	3.05 ± 0.64	0.015*
Bias Detection	3.60 ± 0.39	3.55 ± 0.64	0.705 (n.s)
Readability	3.95 ± 0.16	4.05 ± 0.37	0.317 (n.s)
Natural Language Flow	3.55 ± 0.44	3.45 ± 0.16	0.414 (n.s)
Authenticity of Voice	3.00 ± 0.53	3.00 ± 0.24	1.000 (n.s)
Idiomatic Language	3.20 ± 0.42	2.95 ± 0.37	0.059 (n.s)
Rhetorical Devices	1.75 ± 0.26	1.80 ± 0.26	0.317 (n.s)
Variability & Redundancy	3.50 ± 0.41	3.60 ± 0.32	0.480 (n.s)
Contextual Coherence	3.85 ± 0.63	3.25 ± 0.35	0.043*
Subtlety	3.60 ± 0.78	3.15 ± 0.63	0.037*
Citation Accuracy	1.90 ± 1.05	2.30 ± 1.51	0.339 (n.s)
Evidence Integration	2.40 ± 0.57	1.65 ± 0.67	0.027*

*significant (p < 0.050); n. s.: p > 0.050.

SD, standard deviation.

Across the seven binary criteria, both models consistently used references on every task with no criterion reaching significance in Holm-corrected McNemar tests (Table 3).

**Table 3.** Count of “yes”-ratings and statistical comparison between DeepSeek-R1 and ChatGPT-o1 across all evaluation criteria for Binary-based evaluations for both Essays and Q&A using McNemar Test with Holm-Correction (n = 7 rating criteria, n = 5 Essays, n = 5 Q&As, n = 2 rater).

Rating criteria	DeepSeek-R1	ChatGPT-o1	McNemar (Holm-corrected)
	Counts of yes (in %)	Counts of yes (in %)	P-value
References used	10 (100%)	10 (100%)	1.000 (n.s.)
High-quality Reference	3 (30%)	4 (40%)	1.000 (n.s.)
Overall Preference	2 (20%)	1 (10%)	1.000 (n.s.)
Word Limit respected	9 (90%)	5 (50%)	0.221 (n.s.)
AI Authorship Indicated	1 (10%)	5 (50%)	0.134 (n.s.)
Factual Evidence Verification	2 (20%)	4 (40%)	0.480 (n.s.)
Academic/Clinical Suitability	4 (40%)	5 (50%)	1.000 (n.s.)

*significant (p < 0.050); n. s.: p > 0.050.

Stratified analyses by question format (Supplementary Tables S4, S5) revealed a consistent pattern: in essays, both models performed comparably across all criteria (all p ≥ 0.25), whereas in Q&A tasks DeepSeek-R1 displayed more pronounced advantages, with Nuance and Contextual Coherence reaching the minimum achievable exact p-value at n = 5 pairs (p = 0.0625 each). As these stratum-level p-values do not reach conventional significance, stratified results are interpreted descriptively. Per-task mean scores (Supplementary Table S8) show a consistent directional pattern across tasks, with DeepSeek-R1 receiving higher mean ratings in 5 of 10 tasks, ChatGPT-o1 in none, and 5 tasks tied within ±0.2 mean rating points, suggesting that overall results were not driven by a single outlier task.

Inter-rater reliability varied across Likert criteria (Supplementary Table S6). Citation Accuracy showed the highest agreement (ICC = 0.807). The four criteria with statistically significant differences showed poor agreement: Reasoning Depth (ICC = 0.461), Contextual Coherence (0.135), Subtlety (0.477), and Evidence Integration (0.035). Five criteria showed chance-level agreement (ICC < 0): Readability (−0.392), Natural Language Flow (−0.256), Idiomatic Language (−0.158), Rhetorical Devices (−0.088), and Variability & Redundancy (−0.039). The overall ICC(2,1) of 0.466 may be partially driven by the high agreement on Citation Accuracy (ICC = 0.807). For the 7 binary criteria, Cohen’s κ ranged from 0.105 to 1.000 (overall κ = 0.746; Supplementary Table S7).

## Discussion

Our findings demonstrate that DeepSeek-R1 outperformed ChatGPT-o1 overall for both generating scientific neuroradiology essays and evidence-based answering of short Q&A settings, driven largely by superior Reasoning Depth, Contextual Coherence, Subtlety, and Evidence Integration. However, both models displayed low performance over most criteria, particularly in referencing - this may not only reflect difficulties in citation accuracy, but also broader deficits in analytical depth, structured reasoning, and the ability to integrate and synthesize clinical and scientific evidence. These results have important implications for the deployment of LLMs in medical research domains. Prior studies have mostly discussed potential applications and ethical implications of LLMs for scientific writing in medicine overall and – when performance was measured – analyzed often only one (typically proprietary) LLM.^12–17^ To our knowledge this study displays the first comprehensive LLM comparison for scientific writing with a focus on neuroradiology.

### Performance of DeepSeek-R1 versus ChatGPT-o1

Across 14 Likert-scale criteria encompassing reasoning depth, contextual coherence, subtlety, evidence integration, and other dimensions of academic writing, DeepSeek-R1 achieved a higher overall mean rating score than ChatGPT-o1. Notably, DeepSeek-R1 showed statistically significant advantages in Reasoning Depth, Contextual Coherence, Subtlety, and Evidence Integration. Given the limited inter-rater agreement for these criteria, these findings are interpreted as exploratory. The performance gap in our study may be partially explained by different training strategies. ChatGPT-o1 development and training remains mostly undisclosed, whereas DeepSeek-R1 combines supervised fine-tuning, reinforcement learning, and iterative refinement.^18^ This approach may support more structured reasoning and could therefore in particular explain the performance differences in *Reasoning Depth* and *Contextual Coherence*, which are both important for addressing complex, domain-specific tasks in scientific writing in neuroradiology.

DeepSeek-R1 produced more concise and better-structured text. For instance, in the essay on MRI for neurodegenerative disease detection, ChatGPT-o1 partially resorted to more informal language and repeated foundational MR principles, while DeepSeek-R1 delivered more concise phrasing and stayed more task-focused. Across the Q&A items, DeepSeek-R1 delivered more refined and targeted answers, for example, its response on current acute stroke imaging guidelines incorporated findings from the DAWN^19^ and DEFUSE^20^ randomized trials.

In the Q&A “MRI Guidelines for Diagnosing Multiple Sclerosis”, ChatGPT-o1 failed to address the McDonald criteria despite their explicit inclusion in the prompt, whereas DeepSeek-R1 provided a focused overview as well as incorporated guidance from both the European Academy of Neurology (EAN) and the Magnetic Resonance Imaging in MS (MAGNIMS) network.

However, both models fabricated hallucinated references so richly detailed that they likely exploit fluent reader bias, underscoring the need for integrated citation‐verification mechanisms. Interestingly, for well-documented topics such as stroke imaging, the models’ cited references were relatively more likely to correspond to real publications, yet they still often failed to follow correct APA formatting or contained inaccurate details.

Despite routine inclusion of references by both models, correct citation rates were remarkably low. In an academic medicine context, false citations can mislead readers and undermine the credibility of generated content. Consequently, any implementation of LLMs in scientific writing workflows must incorporate rigorous citation verification. Despite its overall academic quality advantage, DeepSeek-R1 displayed a substantially higher confirmed fabrication rate, with DOIs consistently resolving to unrelated publications, a failure mode not fully captured by holistic quality ratings and potentially more hazardous than unverifiable citations.

Although DeepSeek-R1 is often prone to lengthy, repetitive outputs,^8,21^ it respected the prescribed word limit in every instance but one, whereas ChatGPT-o1 met that limit in only half of the cases - an inconsistency that can place a significant burden on researchers closely supervising AI-generated content.

When differentiating between essay-style and Q&A tasks, additional nuances become evident. In essays, both models performed comparably, with only minor stylistic differences such as phrasing, coherence, and redundancy. By contrast, in Q&A tasks, DeepSeek-R1 more consistently demonstrated advantages in reasoning depth, nuance, and contextual integration, whereas ChatGPT-o1 showed only marginal benefits in readability and citation accuracy. These observations suggest that essay-based tasks predominantly expose stylistic variation, while Q&A tasks more clearly highlight substantive differences in clinical reasoning and evidence use.

All evaluation criteria were treated as equally weighted, as no validated weighting framework for LLM outputs in academic medicine currently exists. In practice, however, most users are likely to prioritize factual accuracy and citation reliability over stylistic dimensions such as rhetorical devices or authenticity of voice, domains that also carry greater inter-rater subjectivity. Notably, both models performed poorly on Citation Accuracy and Evidence Integration, suggesting that factual unreliability is a shared limitation regardless of weighting scheme. Future studies should engage multi-stakeholder panels to derive empirically grounded criterion weights.

While recent benchmarking suggests that DeepSeek-R1’s reasoning advantages may extend across medical domains,^21^ validation in disciplines with fundamentally different writing demands, such as medical ethics or basic science, remains necessary before broader conclusions can be drawn.

### Inter-rater reliability

Inter-rater reliability varied considerably across criteria, reflecting the inherent subjectivity of writing assessment. Citation Accuracy was the only criterion reaching acceptable agreement (ICC = 0.807). The four criteria with statistically significant differences (Reasoning Depth, Contextual Coherence, Subtlety, and Evidence Integration) displayed poor agreement (ICC range: 0.035–0.477) and conclusions from these comparisons are accordingly treated as exploratory throughout this discussion. For the five stylistic criteria with chance-level agreement, findings are reported descriptively only and no comparative conclusions are drawn. Binary criteria showed substantial overall agreement (κ = 0.746), with the exception of AI Authorship Indicator (κ = 0.105). Joint development of the evaluation instrument by both raters may additionally affect the independence assumptions underlying ICC and Cohen’s κ.

### Open-source versus proprietary models

Open-source models like DeepSeek-R1 offer transparency, local deployment, and the ability to tailor the model to sensitive healthcare data, though they demand more in-house technical expertise. Its public weights and modest hardware needs enable institutions to run and refine the model on-site, but undisclosed training data and code leave questions about provenance, reproducibility, and governance.^8^ Furthermore, our results highlight the importance of assessing how open-source LLMs affect global healthcare equity. While on-site, open-source models can lower costs and aid under-resourced regions – however, uneven access to high-performance hardware and quality data risks deepening the digital divide.^22^ Future work should investigate collaborative open-source networks, local deployments, and cost-efficient adaptations – and establish policy, international partnerships, and resource-sharing to ensure LLMs enhance global health and academic equity.

### Implications for academic and institutions

Our findings underscore that open-source LLMs hold significant promise compared to proprietary systems. Models like DeepSeek-R1 can be fully audited, locally deployed, and iteratively fine-tuned on institution-specific datasets, showing directional gains in coherence and reasoning depth. For complex, domain-specific applications such as scientific writing in neuroradiology, academic and clinical institutions should therefore consider supporting open-source model development and fine-tuning pipelines facilitating reproducible research and foster collaborative enhancement by the scientific community.

Due to the potential risks of using LLMs in scientific research, academic and clinical institutions should prioritize transparent governance, implement rigorous verification pipelines such as human-in-the-loop review and retrieval-augmented fact-checking, and establish clear AI literacy and ethical guidelines. Implementing these safeguards can help prevent hallucinations, citation errors, and authorship concerns while preserving the integrity of scholarly work.

### Limitations

This study has several limitations. First, tasks were limited to neuroradiology topics and two writing formats. Replication across other medical disciplines and academic writing genres is needed to establish broader validity. Second, with just two expert raters who co-developed the evaluation instrument, independence assumptions of ICC and Cohen’s κ may be affected, individual bias cannot be excluded, and the multidimensional rating scale, while comprehensive, remains inherently subjective. The same two authors (S.E., J.N.) also served as raters, which may introduce confirmation and anchoring bias despite full a priori blinding. Inter-rater reliability was limited for most Likert criteria, and generalizability remains accordingly constrained. Future studies should employ raters fully independent of instrument development, ideally including non-clinical domain experts for topic-specific sub-criteria. Third, although DeepSeek-R1 outperformed ChatGPT-o1 on key metrics, both models’ absolute scores indicate room for improvement, particularly in evidence integration. Fourth, with only ten tasks, the study was powered to detect large effects only (minimum detectable dz ≈ 1.0 at 80% power). After Holm correction, no individual criterion retained significance. Effect sizes should therefore be considered alongside p-values when interpreting observed differences. Fifth, the absence of a human-authored control group means that the low detectability of AI-generated markers should not be interpreted as evidence of indistinguishability from expert human writing, but rather as inconsistent recognition of AI stylistic features within a pairwise LLM-only evaluation. A prospective comparison with prompt-matched human-authored texts remains an important next step. Sixth, the academic tasks covered a heterogeneous range of topics, which supports content validity but implies varying performance expectations across topics. Stratified analyses suggest that no single task drove the overall conclusions. Future work should employ more homogeneous question groupings to enable cleaner comparisons. Seventh, one of the evaluated models (ChatGPT-o1) was also used for preliminary topic scoping during study design. Although final topic selection was performed independently by the authors and all topics correspond to established neuroradiology subject areas, future studies should employ fully LLM-independent prompt curation. Last, ongoing LLM advancements could limit the relevance of our findings for subsequent model versions.

### Future directions

Future work should increase the diversity of writing tasks, including more complex case reports and grant proposals. Integrating retrieval-augmented generation and automated citation‐verification pipelines could help mitigate hallucinations and referencing errors, while human-in-the-loop studies would clarify optimal workflows for clinician × LLM collaboration. Longitudinal analyses of model updates and comparisons with emerging open-source and proprietary systems will be essential for understanding performance drift over time. Moreover, LLMs fine-tuned on specialized research datasets need targeted evaluation to quantify the benefits of domain adaptation. Finally, user studies assessing real‐world usability, trust, and impact on research efficiency will inform best practices for responsibly embedding LLMs into academic and clinical environments. Larger-scale evaluations with substantially expanded task corpora across diverse academic writing formats (e.g., systematic reviews, case reports) will be needed to confirm and refine the present findings.

## Conclusion

In this controlled evaluation of academic writing tasks in neuroradiology, both tested models produced outputs with reasonable academic quality and fluent linguistic expression, but frequent inaccuracies and unreliable references highlighted clear limitations in factual reliability. The open-source model DeepSeek-R1 achieved higher overall ratings than the proprietary ChatGPT-o1, particularly in reasoning depth, coherence, subtlety, and evidence integration, demonstrating the potential of open-source solutions for specialized scientific writing. This performance advantage, together with the transparency and adaptability inherent to open-source approaches, underscores their value for the responsible integration of LLMs into academic medicine. To align with good scientific practice, however, robust verification and quality control pipelines remain essential to safeguard against hallucinations and citation errors.

## Supplemental material

Supplemental material -Open-source versus proprietary large language models for academic writing in medicine: A controlled evaluation of DeepSeek-R1 and ChatGPT-o1 in neuroradiology Supplemental material for Open-source versus proprietary large language models for academic writing in medicine: A controlled evaluation of DeepSeek-R1 and ChatGPT-o1 in neuroradiology by Semil Eminovic, Bogdan Levita, Robin Schmidt, Maximilian Lindholz, Dmitriy Desser, Tobias Penzkofer, Mike P. Wattjes and Jawed Nawabi in DIGITAL HEALTH.

## Data Availability

Data generated or analyzed during the study are available from the corresponding author by request.*

## Appendix

### List of abbreviations

AHA: American Heart Association
AI: Artificial intelligence
APA: American Psychological Association (referencing style)
ASA: American Stroke Association
CT: Computed tomography
CTA: Computed tomography angiography
CTP: Computed tomography perfusion
DOI: Digital object identifier
DWI: Diffusion-weighted imaging
EAN: European Academy of Neurology
EANO: European Association of Neuro-Oncology
EU: European Union
FDG-PET: Fluorodeoxyglucose positron emission tomography
FLAIR: Fluid-attenuated inversion recovery
fMRI: Functional magnetic resonance imaging
ICC: Intraclass correlation coefficient
LLM: Large language model
MAGNIMS: Magnetic resonance Imaging in Multiple Sclerosis (network)
MR: Magnetic resonance
MRI: Magnetic resonance imaging
MS: Multiple sclerosis
NIA-AA: National Institute on Aging–Alzheimer’s Association
n.s.: Not significant
PET: Positron emission tomography
PET/MRI: Positron emission tomography/magnetic resonance imaging
PMID: PubMed identifier
Q&A: Question and answer
SD: Standard deviation
T1: T1-weighted (imaging).
