# Supplementary material for: Open-source versus proprietary large language models for academic writing in medicine: A controlled evaluation of DeepSeek-R1 and ChatGPT-o1 in neuroradiology
Source: Digit Health. 2026 Sep 2;12:20552076261464745. doi: 10.1177/20552076261464745 (PMC13539036; doi:10.1177/20552076261464745)
Supplement: Supplementary file 1 [file sj-pdf-1-dhj-10.1177_20552076261464745.pdf]

Supplementary Material

Supplementary Text 1 All five prompts for the Essay Writing Task

1) <u>'AI-Supported MRI Analysis for Detecting Neurodegenerative Diseases'</u>

Write a scientific essay (around 1000 words) on the topic: 'AI-Supported MRI Analysis for Detecting Neurodegenerative Diseases'. Structure the essay into an introduction, main body, and conclusion. Explain the role of AI in detecting Alzheimer's, Parkinson's, and Multiple Sclerosis. Analyze recent studies (from 2020 onwards) and discuss benefits and ethical challenges. Use factual, scientific language and cite relevant sources in APA style. Ensure that the text has a logical structure and that the arguments are supported by examples.

2) <u>'Radiomics and Machine Learning in Brain Tumor Diagnosis'</u>

Write a scientific essay (around 1000 words) on the topic: 'Radiomics and Machine Learning in Brain Tumor Diagnosis'. Structure the essay into an introduction, main body, and conclusion. Explain the technical foundations of radiomics, present recent research findings (from 2020 onwards), and discuss the clinical impact. Address challenges such as data quality, interpretability, and algorithmic bias. Use factual, scientific language and cite relevant sources in APA style. Ensure that the text has a logical structure and that the arguments are supported by examples.

3) <u>'The Role of Functional MRI (fMRI) in Pre-surgical Epilepsy Diagnosis'</u>

Write a scientific essay (around 1000 words) on the topic: 'The Role of Functional MRI (fMRI) in Pre-surgical Epilepsy Diagnosis'. Structure the essay into an introduction, main body, and conclusion. Describe the importance of fMRI, present recent research (from 2020 onwards), and discuss advantages and limitations compared to other methods. Use factual, scientific language and cite relevant sources in APA style. Ensure that the text has a logical structure and that the arguments are supported by examples.

4) <u>'Comparison between CT perfusion imaging and MRI in ischemic stroke'</u>

Write a scientific essay (around 1000 words) on the topic: 'Comparison between CT perfusion imaging and MRI in ischemic stroke'. Structure the essay into an introduction, main body, and conclusion. Describe the technical differences, present recent research findings (from 2020 onwards), compare sensitivity and specificity of both techniques and cite current guidelines (e.g. AHA 2023). Use factual, scientific language and cite relevant sources in APA style. Ensure that the text has a logical structure and that the arguments are supported by examples.

5) <u>'Ethical and data protection challenges of AI in neuroradiology'</u>

Write a scientific essay (around 1000 words) on the topic: “Ethical and data protection challenges of AI in neuroradiology”. Structure the essay into an introduction, main body, and conclusion. Discuss topics such as data protection, bias, explainability and legal frameworks. Include recent research findings (from 2020 onwards) and EU directives (e.g. EU AI Act). Use factual, scientific language and cite relevant sources in APA style. Ensure that the text has a logical structure and that the arguments are supported by examples.

Supplementary Text 2 All five prompts for the Q&A Writing Task

1) Current Guidelines for Imaging in Acute Stroke Diagnosis

Answer the following evidence-based question:

Question: What are the current guidelines for imaging in acute stroke diagnosis according to the American Heart Association (AHA) and American Stroke Association (ASA) or comparable professional societies?

Answer requirements:
- State the recommended imaging modalities and their purpose (e.g., CT, CTA, CTP, MRI).
- Describe the indications for perfusion imaging and large vessel occlusion detection.
- Mention the time windows for different imaging methods (e.g., early CT vs. advanced MRI for late presentation).
- Refer to the latest guidelines.
- Summarize your answer in a maximum of 200 words.
- Cite the source (e.g., AHA guidelines, year) in APA style.

2) MRI Guidelines for Diagnosing Multiple Sclerosis

Answer the following evidence-based question and pay attention to the answer requirements:

Question: What are the current guidelines for MRI diagnostics in multiple sclerosis (MS) according to the European Academy of Neurology (EAN) or comparable professional societies?

Answer requirements:
- State the recommended MRI sequences and their purpose (e.g., FLAIR, DWI, T1 with contrast).
- Describe the recommended intervals for follow-up examinations.
- State specific diagnostic criteria (e.g., McDonald criteria 2017).
- Refer to the latest guidelines.
- Summarize your answer in a maximum of 200 words.
- Cite the source (e.g., EAN guidelines, year) in APA style.

3) Indications for MR Spectroscopy in Brain Tumor Diagnostics

Answer the following evidence-based question and pay attention to the answer requirements:

Question: When is MR spectroscopy recommended for the evaluation of brain tumors according to the European Association of Neuro-Oncology (EANO) or comparable professional societies?

Answer requirements:
- State the clinical indications for using MR spectroscopy (e.g., differentiating tumor from radiation necrosis).
- Describe key spectroscopic biomarkers (e.g., Choline/N-acetylaspartate ratio).
- Mention situations where spectroscopy adds diagnostic value beyond standard MRI.
- Refer to the latest guidelines.

- Summarize your answer in a maximum of 200 words.
- Cite the source (e.g., EANO guidelines, year) in APA style.

4) DWI (Diffusion-Weighted Imaging) Recommendations for Stroke Diagnosis

Answer the following evidence-based question and pay attention to the answer requirements:

Question: What are the current recommendations for diffusion-weighted imaging (DWI) in stroke diagnosis according to the American Heart Association (AHA) or comparable professional societies?

Answer requirements:
- State the role of DWI in detecting acute ischemic stroke.
- Describe the advantages of DWI over conventional MRI sequences.
- Mention how DWI is used for detecting early infarcts and penumbra regions.
- Refer to the latest guidelines.
- Summarize your answer in a maximum of 200 words.
- Cite the source (e.g., AHA guidelines, year) in APA style.

5) PET/MRI Recommendations for Alzheimer's Disease Diagnosis

Answer the following evidence-based question and pay attention to the answer requirements:

Question: What are the current recommendations for PET/MRI in Alzheimer's disease diagnosis according to the National Institute on Aging–Alzheimer's Association (NIA-AA) or comparable professional societies?

Answer requirements:
- State the role of PET/MRI in Alzheimer's disease diagnosis (e.g., amyloid-PET for plaque detection, FDG-PET for hypometabolism).
- Describe when PET/MRI is indicated (e.g., in cases with atypical presentations or for differential diagnosis).
- Mention complementary imaging biomarkers (e.g., hippocampal atrophy on MRI).
- Refer to the latest guidelines.
- Summarize your answer in a maximum of 200 words.
- Cite the source (e.g., NIA-AA guidelines, year) in APA style.

Supplementary Table 1. Overview of Evaluation Criteria with Definitions and Anchored Likert Scale as well as Binary Scale Descriptions Used for Radiologist Assessment.

Criteria	Definition	Likert-Scale
Academic Quality	Is the content well-	1 = Poor: Disorganized, unclear, and poorly

	structured, clear, and academically rigorous?	substantiated<br>2 = Fair: Somewhat structured but lacks clarity and coherence<br>3 = Good: Generally well-organized with minor readability issues<br>4 = Very Good: Clear, well-structured, and easy to follow<br>5 = Excellent: Exceptionally well-organized, clear, and academically rigorous
Nuance	Does the response capture subtle distinctions and context?	1 = Poor: Oversimplified, lacks depth, and ignores important distinctions<br>2 = Fair: Some awareness of nuance but still overly generalized<br>3 = Good: Recognizes key nuances but could explore them further<br>4 = Very Good: Demonstrates strong contextual understanding<br>5 = Excellent: Captures complex distinctions with precision
Reasoning Depth	Does the AI provide logical and well-supported arguments?	1 = Poor: Lacks logic, coherence, or evidence-based reasoning<br>2 = Fair: Some reasoning present but weakly supported<br>3 = Good: Provides logical arguments with moderate support<br>4 = Very Good: Well-reasoned with strong evidence and justification<br>5 = Excellent: Deep, critical analysis with robust supporting evidence
Bias Detection	Does the AI demonstrate neutrality in medical	1 = Poor: Strong bias or misinformation present<br>2 = Fair: Some bias or unbalanced perspectives

	discussions?	3 = Good: Generally neutral, with minor issues<br>4 = Very Good: Mostly unbiased and balanced in approach<br>5 = Excellent: Completely neutral, considers multiple perspectives fairly
Readibility	Is the content accessible to a clinically trained reader with working knowledge of neuroimaging terminology?	1 = Poor: Difficult to understand, overly technical, or confusing<br>2 = Fair: Somewhat understandable but still challenging<br>3 = Good: Readable with occasional complexity<br>4 = Very Good: Clear and easy to comprehend<br>5 = Excellent: Exceptionally well-written and highly accessible
Human-Likeness & Naturalness		
Natural Language Flow	How naturally and fluidly does the text read, with varied sentence structures and smooth transitions?	1 = Completely mechanical and stilted<br>2 = Somewhat formulaic with limited variation<br>3 = Moderately natural with occasional robotic phrasing<br>4 = Mostly natural, showing varied sentence structures<br>5 = Exceptionally natural and fluid, indistinguishable from human writing
Authenticity of Voice	To what extent does the writing convey a distinct personal voice rather than a generic, standardized tone?	1 = Lacks any personal voice; very generic<br>2 = Barely any individuality; overly standardized<br>3 = Some traces of personal style but still mostly uniform<br>4 = Noticeable voice with personal touches and slight imperfections<br>5 = Rich, distinctive, and consistently human-like in tone
Use of Idiomatic and Context-Specific Language	How effectively does the text integrate idiomatic expressions and domain-specific language to feel	1 = Extremely literal and devoid of idiomatic expressions<br>2 = Rare use of colloquial language; overly formal<br>3 = Adequate use of context-specific expressions, but

	natural and context-aware?	sometimes generic<br>4 = balance of idiomatic expressions and domain-specific language<br>5 = Exceptionally well-integrated, natural idioms and contextual references
Stylistic and Rhetorical Complexity		
Rhetorical Devices & Emotional Nuance	Does the writing employ metaphors, analogies, or subtle emotional cues to enrich its rhetorical impact?	1 = Completely flat, no rhetorical flair or emotional cues<br>2 = Minimal use of rhetorical devices; lacks nuance<br>3 = Some use of metaphors, analogies, or emotional cues, but inconsistent<br>4 = Effectively employs rhetorical devices and subtle emotional cues<br>5 = Masterfully uses metaphors, analogies, and nuanced emotion in a way that feels authentic
Variability & Redundancy	How diverse are the sentence structures and word choices, and does the text avoid noticeable repetition?	1 = Highly repetitive, with no variation in expression<br>2 = Some repetition; overall variation is limited<br>3 = Moderate variation; some repeated phrases still present<br>4 = Good variety in sentence structures and word choice<br>5 = Exceptionally varied with no noticeable repetitive patterns
Semantic Coherence & Adaptability		
Contextual Adaptation & Coherence	Is the content logically coherent and well-adapted to its context, with a clear, progressive flow of ideas?	1 = Lacks coherence; does not adapt to context<br>2 = Some coherence but struggles with context-specific details<br>3 = Generally coherent; minor issues in adapting to nuance<br>4 = Strong contextual adaptation with clear, logical progression<br>5 = Exceptionally coherent and context-aware,

		demonstrating deep understanding of the subject
Subtlety in Argumentation	Does the argument capture nuanced distinctions and present a sophisticated, layered perspective?	1 = Oversimplified; fails to capture important nuances<br>2 = Minimal nuance; remains overly generalized<br>3 = Recognizes key nuances but could explore them more deeply<br>4 = Demonstrates strong contextual understanding with nuanced distinctions<br>5 = Captures complex distinctions with precision and depth
Citation and Reference Authenticity		
Citation Accuracy	Are all citations and reference details correct, complete, and free from hallucinated or incorrect information?	1 = Citations are clearly hallucinated or completely incorrect<br>2 = Many errors in DOIs, publication details, or authorship<br>3 = Some citations are accurate, but several inconsistencies persist<br>4 = Most citations are accurate with only minor errors<br>5 = All citations and references are accurate, complete, and seamlessly integrated
Evidence Integration	How thoroughly and accurately does the text integrate supporting evidence to substantiate its claims?	1 = Claims are unsupported or entirely inaccurate<br>2 = Minimal evidence support; references are unreliable<br>3 = Moderate support with some evidence, but inconsistencies exist<br>4 = Well-integrated evidence, though occasional inaccuracies may be present<br>5 = Robust, evidence-based arguments fully supported by accurate references
Binary Ratings		
Evidence-based References	Are the claims supported by evidence or references?	1 = Yes - the response contains factually accurate information, supported by evidence or references<br>0 = No - The response contains factual inaccuracies, lacks evidence, or misrepresents information

Quality of References	If supported by correct evidence, ist the reference / source high quality? (e.g. peer-reviewed, official guidelines)?	1 = Yes - The source is of high quality (peer-reviewed, official guidelines, academic publications, etc.).<br>0 = No - The source is not of high quality (e.g. blogs, questionable websites).
Overall Preference	Which AI-generated response do they prefer?	1 = The response from LLM1 is preferred over the response from LLM2<br>0 = The response from LLM2 is preferred over the response from LLM1
Word Limit	Was the word limit respected?	1 = The word limit was respected<br>0 = The word limit was not respected
Overall binary evaluation		
AI Authorship Indicator	Does the text exhibit stylistic or mechanical markers of AI authorship? Rated 'Yes' if formulaic, vague, or repetitive phrasing patterns typical of AI-generated text were identified (Gao et al. 2023; Casal & Kessler, 2023).	1 = Yes, if stylistic or mechanical markers are evident,<br>0 = No, if it appears authentically human
Factual Evidence Verification	Is every piece of evidence provided factually correct and verifiable?	1 = Yes if all references and metrics are accurate<br>0 = No if they are hallucinated or incorrect
Academic/Clinical Suitability	Is the text appropriate and sufficiently rigorous for academic or clinical use?	1 = Yes, it meets the standards of academic rigor and evidence support<br>0 = Noif it does not it meets the standards of academic rigor and evidence support

Legend

- *Academic Quality*: Rigor is operationalized as: (i) logical consistency of argumentation, (ii) appropriate qualification of claims (evidence vs. inference), and (iii) acknowledgement of uncertainty where applicable. These components reflect the intended meaning as applied during rating.
- *Readability*: Although this target audience was not stated in the prompts, it served as the a priori evaluative reference for both raters; ratings reflect this implicit standard rather than a model-specific adaptation.
- *AI Authorship Indicator*: Applied markers included formulaic transitional phrasing (e.g., 'It is important to note', 'Moreover', 'Furthermore'), redundant restatement of the prompt, excessive parallel sentence constructions, over-frequent parenthetical qualifications, and hedging phrases atypical of academic medical writing. No validated taxonomy of AI stylistic markers currently exists. Inter-rater agreement was accordingly low (κ = 0.105).

Supplementary Table S2. Granular per-reference citation analysis by model and task type.

**Model**	**Task Type**	**n**	**Fully verifiable n (%)**	**Partially correct n (%)**	**Unverifiable n (%)**	**Fabricated n (%)**
ChatGPT-o1	Essays	34	5 (14.7)	3 (8.8)	25 (73.5)	1 (2.9)
ChatGPT-o1	Q&A	5	3 (60.0)	2 (40.0)	0 (0.0)	0 (0.0)
ChatGPT-o1	**Total**	**39**	**8 (20.5)**	**5 (12.8)**	**25 (64.1)**	**1 (2.6)**
DeepSeek-R1	Essays	42	10 (23.8)	11 (26.2)	3 (7.1)	18 (42.9)
DeepSeek-R1	Q&A	7	1 (14.3)	5 (71.4)	1 (14.3)	0 (0.0)
DeepSeek-R1	**Total**	**49**	**11 (22.4)**	**16 (32.7)**	**4 (8.2)**	**18 (36.7)**

Legend. (a) Fully verifiable: correct authors, title, journal, year, and DOI/PMID. (b) Partially correct: real publication with ≥1 incorrect bibliographic detail. (c) Unverifiable: not locatable via PubMed, Google Scholar, or DOI lookup. (d) Fabricated: DOI resolving to a different publication or equivalent positive evidence of hallucination. Overall category distribution: χ^2=35.98, df=3, p<0.001. Fabrication rate (d): Fisher's exact p<0.001. Fully verifiable rate (a): Fisher's exact p=1.00.

Supplementary Table S3 Wilcoxon signed-rank test results including unadjusted and Holm-corrected p-values and effect sizes for all 14 Likert criteria (n = 10 pairs).

Rating Criteria	p (unadjusted)	p (Holm-corrected)	r_rb	Interpretation
Academic Quality	0.380	1.000	0.36	n.s.
Nuance	0.065	0.588	0.72	n.s.
Reasoning Depth	0.015	0.213	1.00	n.s.
Bias Detection	0.705	1.000	0.20	n.s.
Readability	0.317	1.000	−0.50	n.s.
Natural Language Flow	0.414	1.000	0.33	n.s.
Authenticity of Voice	1.000	1.000	0.00	n.s.
Idiomatic Language	0.059	0.588	0.71	n.s.
Rhetorical Devices	0.317	1.000	−1.00	n.s.
Variability & Redundancy	0.480	1.000	−0.33	n.s.
Contextual Coherence	0.043	0.478	0.90	n.s.
Subtlety	0.037	0.449	0.86	n.s.
Citation Accuracy	0.339	1.000	−0.43	n.s.
Evidence Integration	0.027	0.355	0.78	n.s.

r_rb = matched-pairs rank-biserial correlation; positive values favor DeepSeek-R1. All Holm-corrected p-values > 0.05 (n.s.).

Supplementary Table S4. Mean ratings and stratified comparison between DeepSeek-R1 and ChatGPT-o1 across all evaluation criteria for Likert-based evaluations for Essays only, including unadjusted and Holm-corrected exact p-values and effect sizes (n = 14 rating criteria, n = 5 pairs, n = 2 raters).

**Rating Criteria**	**DeepSeek-R1 Mean ± SD**	**ChatGPT-o1 Mean ± SD**	**W**	**p (unadj.)**	**p (Holm)**	**r_rb**
Overall (all criteria)	2.85 ± 0.24	2.76 ± 0.16	—	—	—	—
Academic Quality	3.30 ± 0.27	3.50 ± 0.35	2.5	0.625	1.000	−0.500
Nuance	2.70 ± 0.27	2.80 ± 0.45	2.0	1.000	1.000	−0.333

Reasoning Depth	3.00 ± 0.35	2.50 ± 0.35	0.0	0.250	1.000	1.000
Bias Detection	3.30 ± 0.27	3.10 ± 0.65	2.0	1.000	1.000	0.333
Readability	3.90 ± 0.22	3.80 ± 0.27	0.0	1.000	1.000	1.000
Natural Language Flow	3.40 ± 0.42	3.40 ± 0.22	1.5	1.000	1.000	0.000
Authenticity of Voice	2.80 ± 0.57	3.10 ± 0.22	0.0	0.500	1.000	−1.000
Idiomatic Language	3.20 ± 0.57	3.00 ± 0.50	2.5	0.625	1.000	0.500
Rhetorical Devices	1.50 ± 0.00	1.60 ± 0.22	0.0	1.000	1.000	−1.000
Variability & Redundancy	3.30 ± 0.45	3.70 ± 0.45	0.0	0.250	1.000	−1.000
Contextual Coherence	3.30 ± 0.27	3.40 ± 0.42	0.0	1.000	1.000	−1.000
Subtlety	2.90 ± 0.22	2.70 ± 0.57	1.5	0.750	1.000	0.500
Citation Accuracy	1.20 ± 0.27	1.00 ± 0.00	0.0	0.500	1.000	1.000
Evidence Integration	2.10 ± 0.42	1.10 ± 0.22	0.0	0.063	0.875	1.000

Positive r_rb values favor DeepSeek-R1. For criteria where all pairwise differences equal zero, no test was performed (—). The minimum achievable exact p-value at n = 5 pairs is 0.0625; no criterion reaches conventional significance (α = 0.05) before or after Holm correction.

Legend: SD, standard deviation; W, Wilcoxon signed-rank statistic; r_rb, matched-pairs rank-biserial correlation.

Supplementary Table S5. Mean ratings and stratified comparison between DeepSeek-R1 and ChatGPT-o1 across all evaluation criteria for Likert-based evaluations for Q&A tasks only, including unadjusted and Holm-corrected exact p-values and effect sizes (n = 14 rating criteria, n = 5 pairs, n = 2 raters).

Rating Criteria	DeepSeek-R1 Mean ± SD	ChatGPT-o1 Mean ± SD	W	p (unadj.)	p (Holm)	r_rb
Overall (all criteria)	3.61 ± 0.13	3.28 ± 0.10	—	—	—	—
Academic Quality	4.10 ± 0.42	3.60 ± 0.22	0.0	0.250	1.000	1.000
Nuance	4.50 ± 0.50	3.10 ± 0.22	0.0	0.063	0.813	1.000
Reasoning Depth	4.20 ± 0.45	3.60 ± 0.22	0.0	0.125	1.000	1.000
Bias Detection	3.90 ± 0.22	4.00 ± 0.00	0.0	1.000	1.000	−1.000
Readability	4.00 ± 0.00	4.30 ± 0.27	0.0	0.250	1.000	−1.000
Natural Language Flow	3.70 ± 0.45	3.50 ± 0.00	2.5	0.625	1.000	0.500
Authenticity of Voice	3.20 ± 0.45	2.90 ± 0.22	2.0	0.500	1.000	0.600
Idiomatic Language	3.20 ± 0.27	2.90 ± 0.22	0.0	0.250	1.000	1.000
Rhetorical Devices	2.00 ± 0.00	2.00 ± 0.00	—	—	—	0.000
Variability &	3.70 ± 0.27	3.50 ± 0.00	0.0	0.500	1.000	1.000

Redundancy						
Contextual Coherence	4.40 ± 0.22	3.10 ± 0.22	0.0	0.063	0.813	1.000
Subtlety	4.30 ± 0.27	3.60 ± 0.22	0.0	0.125	1.000	1.000
Citation Accuracy	2.60 ± 1.08	3.60 ± 0.96	1.0	0.250	1.000	−0.800
Evidence Integration	2.70 ± 0.57	2.20 ± 0.45	3.5	0.375	1.000	0.533

Positive r_rb values favor DeepSeek-R1. For criteria where all pairwise differences equal zero, no test was performed (—). The minimum achievable exact p-value at n = 5 pairs is 0.0625; no criterion reaches conventional significance ($\alpha = 0.05$) before or after Holm correction.

Legend: SD, standard deviation; W, Wilcoxon signed-rank statistic; r_rb, matched-pairs rank-biserial correlation.

Supplementary Table S6 Interrater agreement with two-way random-effects, single-measure intraclass correlation coefficient (ICC (2,1)) for Likert-rated criteria across both LLMs

Rating Criteria	ICC(2,1)
Overall	0.466
Academic Quality	0.103
Nuance	0.225
Reasoning Depth	0.461
Bias Detection	0.341
Readability	-0.392
Natural Language Flow	-0.256
Authenticity of Voice	0.034
Idiomatic Language	-0.158
Rhetorical Devices	-0.088
Variability & Redundancy	-0.039
Contextual Coherence	0.135
Subtlety	0.477
Citation Accuracy	0.807

Evidence Integration	0.035

Supplementary Table S7 Interrater agreement with Cohen's κ for binary rating criteria across both LLMs

Rating Criteria	Cohen's κ
Overall	0.746
References used	1.000
High-quality Reference	0.711
Word Limit	1.000
AI Authorship Indicator	0.105
Factual Evidence Verification	0.791
Academic/Clinical Suitability	0.905

Supplementary Table S8. Per-task overall mean Likert scores (averaged across all 14 criteria and both raters) for DeepSeek-R1 and ChatGPT-o1 across all 10 tasks. Δ = DeepSeek-R1 minus ChatGPT-o1 mean score. Tasks are classified as favoring DeepSeek-R1 ($\Delta > 0.2$), favoring ChatGPT-o1 ($\Delta < -0.2$), or tied ($|\Delta| \leq 0.2$).

Task Type	Task	ChatGPT-o1	DeepSeek-R1	Δ	Classification
Essay	AI-Supported MRI Analysis for Detecting Neurodegenerative Diseases	2.68	2.93	+0.25	DeepSeek-R1
Essay	Radiomics and Machine Learning in Brain Tumor Diagnosis	2.71	2.68	−0.04	Tied
Essay	The Role of fMRI in Pre-surgical Epilepsy Diagnosis	2.57	2.57	0.00	Tied
Essay	Comparison between CT Perfusion Imaging and MRI in Ischemic Stroke	2.93	2.89	−0.04	Tied
Essay	Ethical and Data Protection Challenges of AI in Neuroradiology	2.93	3.18	+0.25	DeepSeek-R1
Q&A	Current Guidelines for Imaging in Acute Stroke Diagnosis	3.36	3.43	+0.07	Tied
Q&A	DWI Recommendations for Stroke Diagnosis	3.29	3.79	+0.50	DeepSeek-R1
Q&A	Indications for MR Spectroscopy in Brain Tumor Diagnostics	3.29	3.64	+0.36	DeepSeek-R1
Q&A	MRI Guidelines for Diagnosing Multiple Sclerosis	3.11	3.64	+0.54	DeepSeek-R1
Q&A	PET/MRI Recommendations for	3.36	3.54	+0.18	Tied

	Alzheimer's Disease Diagnosis				

DeepSeek-R1 higher (Δ > 0.2): 5 of 10 tasks; ChatGPT-o1 higher: 0 of 10 tasks; Tied (|Δ| ≤ 0.2): 5 of 10 tasks.

References

Casal, J. E., & Kessler, M. (2023). Can linguists distinguish between ChatGPT/AI and human writing?: A study of research ethics and academic publishing. *Research Methods in Applied Linguistics*, *2*(3), 100068.

Gao, C. A., Howard, F. M., Markov, N. S., Dyer, E. C., Ramesh, S., Luo, Y., & Pearson, A. T. (2023). Comparing scientific abstracts generated by ChatGPT to real abstracts with detectors and blinded human reviewers. *NPJ digital medicine*, *6*(1), 75.
